# The Newcastle Infirmary Resignations

**Published:** 1910-12-24

**Authors:** 


### SPECIAL INSTITUTIONAL ARTICLES.

### THE NEWCASTLE INFIRMARY RESIGNATIONS.

(From a Newcastle Correspondent.)

To hint a fault is as foolish a proceeding now as ever it was in Pope's day. This is, in effect, what will suggest itself to a good many as a general comment on what has recently happened at the Newcastle Infirmary, particulars of which we give elsewhere. If the Admissions Committee of this institution conjectured that the honorary medical officers were actuated by venal motives in their attitude towards candidates for beds, then no imputation should have been given expression to; but, on the contrary, full investigation into the point should have been made, to be followed either by a definite charge or by silence on the subject. To state that " an impression exists " proves nothing, and saves nothing. Out of the imbroglio thus created it is to be hoped that the tact—rather severely tried of late—of the infirmary authorities may find a speedy way.

So much for short general comment. As for the different directions in which discussion of this topic in detail may lead, they are most numerous. Here it is not possible to dwell upon hospital abuse (with its important corollary the interests of the general practitioner); upon the relations between lay and hospital medical officers; upon the question of payment of the latter; and upon some aspects of that measure usually cited as the Workmen's Compensation Act. We have, too, a conviction (which will be fully shared in locally) that the true inwardness of the situation is concerned with something else; something which may be reckoned an indication, if a small one, of the rapid progress of the democratic idea which has moulded so powerfully events in this country during the last half decade.

Elsewhere figures are quoted which show a great widening during that period of the sphere of activity of Newcastle Infirmary. It is due almost exclusively to a large influx of working-men patients, following upon the creditable response of their organisations to the appeal of one of the Vice-Presidents, Sir Riley Lord, for systematic weekly contributions to the funds of the institution. In every organ of the local Press mention has been lately made of allegations that too much use has thus been made of the infirmary, that slight cases have received treatment which could well have been looked after at home by their own medical men, and that, as one of the latter is quoted as observing, " the institution is now half a charity and half a benefit club."

It would occur here to most people that such cases are neither urgent ones nor likely to need a consultation; and conversely that such as do need a consultation are *ipso facto* likely to be serious, and to require prompt admission. This is a side of the question of which as yet little has been heard.

It is an aspect of the subject which might usefully have been put forward at a discussion of the present infirmary topic at a meeting of the Newcastle Labour Representation Committee, reports of which we gather from the *Newcastle Daily Journal* and the *North Mail* of December 14. The tone of this discussion—or, rather, of one contributor to it—was too adverse to the present management of the institution. The hon. secretary, who thought it democratic to forbid the doctors to select patients for admission, said he thought the working men should, in consideration of their contributions, have an active voice in the administration of the infirmary, and that at present their representation amounted to little more than a figurehead. If this be so, there cannot be much in the force of numbers, since we observe that every £10 subscribed confers the right of nominating a governor, and the number of working-men governors thus arising runs into a few hundreds. There can be nothing but praise for the principle of popular support of hospitals; and no sensible man will gainsay that he who pays the piper shall call the tune. But, as is practically recognised in most medical institutions, there should be a large measure of decentralisation of authority when laymen come to deal with technical experts. There has undoubtedly been as much class antagonism aroused by this dispute, as by the former (and much less important) one occurring at this same institution. A despondent view is not, however, indicated. The working man has probably a great deal more respect for education and medical skill than many much more fashionable people. Bitterness and a reactionary spirit on the one side are therefore to be deprecated just as much as brusqueness and domination on the other. If each party were to suffer some impairment of keenness of vision for the other side's weak points the question of forms of procedure would soon cease to trouble, and a businesslike settlement of the problem under discussion might quickly be arrived at without the interests of patients, institution, outside practitioners, and office-holders suffering at all. It cannot be settled either by flattering the doctors or by refusing colliery contributions or by multiplying clerks. The Conference suggested by the House Committee, if it takes place, will clear the air and lead, everyone will hope, to a satisfactory solution.

### THE CAUSES AND PRESENT POSITION OF THE DEADLOCK.

The following would appear to be the facts which explain the present trouble at the Newcastle Infirmary. On June 4, 1908, the Subscriptions Committee prepared a resolution calling attention to the number of cases received from districts where the support of the infirmary was inadequate or altogether lacking, and submitted it to the House Committee. This latter body, which may be taken as *par excellence* the executive authority of the infirmary, thereupon appointed a sub-committee to consider the admission of patients. The annual report for 1908 states: "In order to meet the criticism that many patients are admitted into the hospital without sufficient care being taken to ascertain whether they are such persons as are entitled to receive the benefit of the charity, the House Committee have appointed a sub-committee to consider the admission of patients. The committee have been able to present a report to the House Committee, and have recommended that the system of registration should be reorganised. During the present year they hope to collect figures and information to enable them to recommend some workable scheme which will solve to some extent this extremely difficult question."

The admission figures in the annual report, from which the above passage is quoted, are striking enough, since they show that, whereas 4,899 patients were admitted to the wards in 1906, the total in 1907 was 6,445, and in 1908 7,548. This rate of increase, it may be added, has shown small signs of diminishing lately, while the out-patient attendance grows at a high speed too. Meanwhile the sub-committee, known as the Patients' Admission Committee, considered the returns of admissions to the hospital over a period of six months, and called for a change in the system of admission, and for a staff for the purpose of registering in-patients. It is to be noted that already at the end of last year the honorary medical staff had called the attention of the authorities to the existence of hospital abuse, and that some of its number were co-opted to the Patients' Admission Committee, which was constituted a permanent body and was granted a staff and accommodation for registration of in-patients in the spring of 1910, the new system coming definitely into vogue a little later.

This committee's report of November 3 last—or rather a statement by the chairman—led to the present unfortunate resignation of the whole honorary medical staff. The report dealt with the admissions for July, August, and September 1910, and the classification, as made by the registration officer, Mr. Fletcher, stated that of 2,071 admissions, 1,305 were sent in on local general practitioners' recommendations, 1,014 were sent to specified members of the honorary staff, and only 291 came under the heading of general recommendations to the infirmary. It stated that great congestion existed, since the waiting list of applicants amounted to some hundreds, some of whom had been waiting the best part of a year; and that the rules for admission of patients were in abeyance, each medical officer acting as his own admission officer. The Chairman stated that "an impression" existed that the way to get into the infirmary early was to consult one of the honoraries, and adduced some (hearsay) evidence that an applicant had said he was saving up money in order to get admitted. Thereupon the members of the honorary medical staff, after an unsuccessful objection to this report, "stated that they are practically charged with dishonourable conduct by giving unfair preference in the admission of patients. They are absolutely unanimous in their decision to relinquish office. There is a definite deadlock between the staff and the Admission Committee."

The House Committee, summoned specially on December 15 to consider this letter, resolved that before considering the question of the resignation of the whole medical staff, which it characterised as nothing less than a calamity, a conference should take place between eight members of the House Committee and eight of the honorary medical staff. At the time of writing it is understood that the honorary medical staff has not yet held a meeting to consider this invitation. Needless to say, the current medical needs of the institution are being fully attended to as usual. The position, then, is that the resignations of the staff have not yet been accepted.

